# Supplementary material for: The interactome of intact mitochondria by cross-linking mass spectrometry provides evidence for coexisting respiratory supercomplexes
Source: Mol Cell Proteomics. 2017 Dec 8;17(2):216–32. doi: 10.1074/mcp.RA117.000470 (PMC5795388; doi:10.1074/mcp.RA117.000470)
Supplement: Supplemental Data [file supp_RA117.000470_133922_0_supp_23978_fzffwf.zip › spectra_annotation/new_mito_protein_spectra_annotation/281-1-66-1-57-1.pdf]

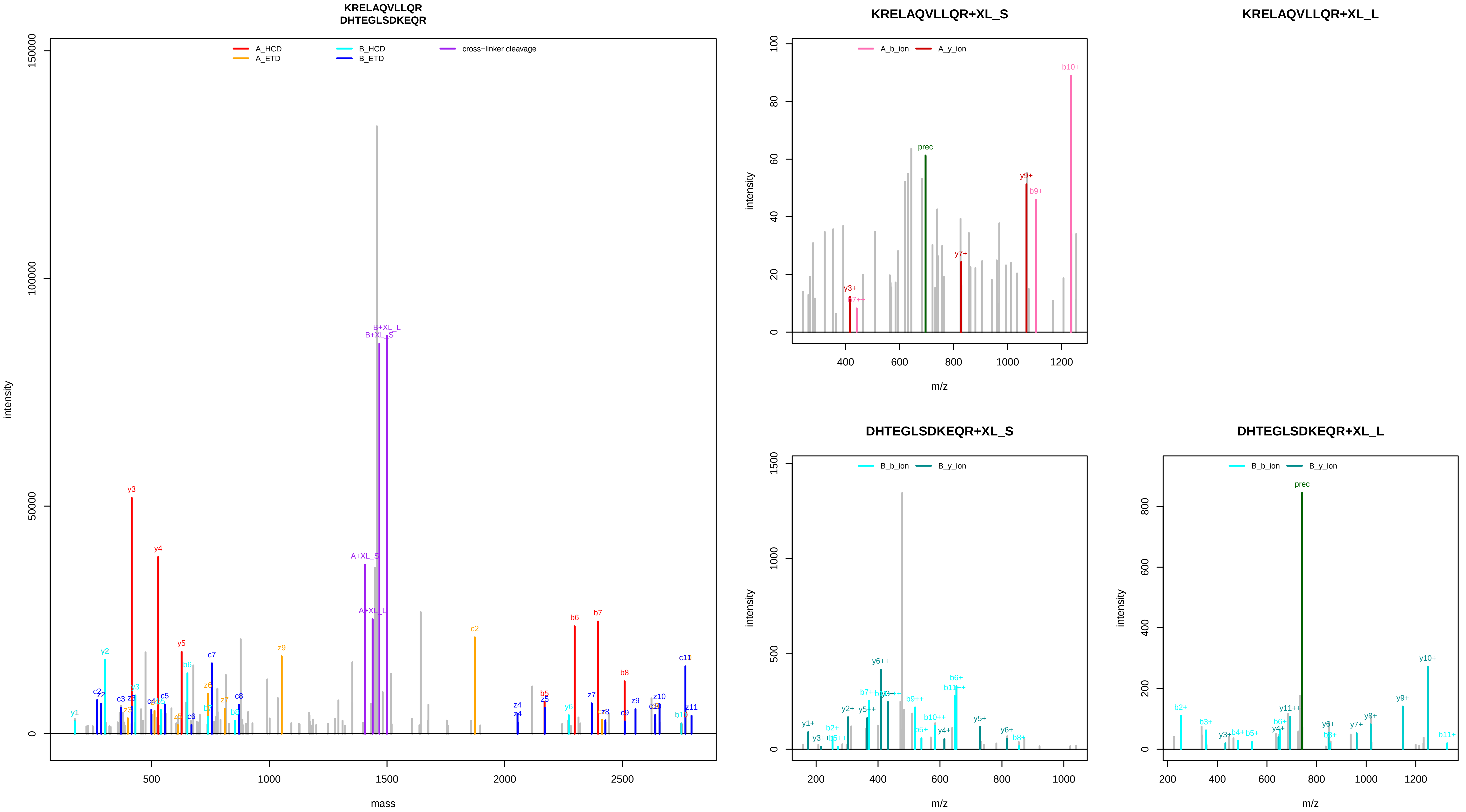
KRELAQVLLQR
DHTEGLSDKEQR
A_HCD
A_ETD
B_HCD
B_ETD
cross−linker cleavage
intensity
mass
B+XL_L
B+XL_S
A+XL_S
A+XL_L
y3
y4
y5
y2
c2
z2
c3
z3
c4
c5
c7
c8
b6
z9
c2
b6
b7
b8
b5
z5
z4
z7
z8
c9
z9
z10
c10
c11
z11
b10
y6
y1
KRELAQVLLQR+XL_S
KRELAQVLLQR+XL_L
A_b_ion
A_y_ion
b10+
prec
y9+
b9+
y7+
y3+
m/z
DHTEGLSDKEQR+XL_S
DHTEGLSDKEQR+XL_L
B_b_ion
B_y_ion
y6++
b6+
b11++
y1+
y2+
y5++
b9++
b10++
y5+
y4+
y6+
b8+
b2+
y3++
prec
y10+
y9+
y8+
y7+
y11++
b3+
b4+
b5+
b11+
